# Ophthalmic acid as a read-out for hepatic glutathione metabolism in humans

**Published:** 2018-03-25

**Authors:** Mierlo Kim MC van, Simon AWG Dello, Mechteld C de Jong, Hans MH van Eijk, Theo M de Kok, Jacob J Briedé, Frank G Schaap, Steven WM Olde Damink, Cornelius HC Dejong

**Affiliations:** ^1^ *Department of Surgery, Maastricht University Medical Center, Maastricht, the Netherlands*; ^2^ *Department of Surgery, Maastricht University, NUTRIM School of Nutrition and Translational Research in Metabolism, Maastricht, the Netherlands*; ^3^ *Department of Toxicogenomics, Maastricht University Medical Center, Maastricht, the Netherlands*; ^4^ *Maastricht University, GROW School for Oncology and Developmental Biology, Maastricht, the Netherlands*; ^5^ *Department of General-, Visceral- and Transplant Surgery, Universitätsklinikum Aachen, Aachen, Germany*; ^6^ *UCL Institute for Liver and Digestive Health, Division of Medicine, London, United Kingdom*

**Keywords:** liver surgery, liver failure, glutathione, ophthalmic acid, acetaminophen

## Abstract

**Background and Aim:**

Animal studies indicated that systemic ophthalmic acid (OPH) is a biomarker for hepatic glutathione (GSH) homeostasis, an important determinant of liver function. We aimed to clarify whether OPH levels can be used as a read-out for hepatic GSH homeostasis after paracetamol (APAP) challenges during pylorus-preserving pancreaticoduodenectomy (PPPD) or partial hepatectomy (PH).

**Methods:**

Nineteen patients undergoing PPPD (n=7, control group) or PH (n=12) were included. APAP (1000 mg) was administered intravenously before resection (first challenge), and six and twelve hours later, with sequential blood sampling during this period. Arterial, hepatic and portal venous blood samples and liver biopsies were taken on three occasions during the first APAP challenge. Plasma and hepatic OPH and GSH levels were quantified, and venous-arterial differences were calculated to study hepatic release.

**Results:**

Systemic GSH levels decreased during the course of the APAP challenge in both surgical groups, without notable change in OPH levels. Hepatic GSH and OPH content was not affected within ˜3 hours after administration of the first APAP dose in patients undergoing PPPD or PH. In this period, net release of OPH by the liver was observed only in patients undergoing PPPD.

**Conclusion:**

The drop in circulating GSH levels following APAP administrations, did not result in an increase in plasma OPH in both patients with an intact liver and in those undergoing liver resection. Hepatic content of GSH and OPH was not affected during the first APAP dose. It is uncertain whether hepatic GSH homeostasis was sufficiently challenged in the present study.

**Relevance for patients:**

In the present study, plasma OPH seemed not useful as a marker for GSH depletion because APAP administration during liver surgery did not lead to (immediate) GSH depletion or increased OPH levels. Based on stable levels of hepatic GSH, OPH and thiyl radicals during surgery, standard APAP administration seems to be safe in a postoperative care program with regards to GSH homeostasis in this specific population. However, no general statements can be made on the basis of the current experiment, since GSH homeostasis and susceptibility to xenobiotic toxicity are influenced by several metabolic and genetic factors.

## 1. Introduction

Prediction of remnant liver function is becoming increasingly important to identify patients at risk of postresectional liver failure [1-3]. One of the important functions of the liver is the defense against diverse forms of (chemical) stress and intoxications [4-6]. For example, radicals are scavenged through reaction with glutathione (GSH), a tripeptide abundantly present in the liver. GSH is synthesized in the cytoplasm, while its degradation by plasma membrane-associated ectoenzymes takes place in the extracellular compartment. The liver releases GSH in bile and sinusoidal blood, and is considered to be the predominant source of GSH in the circulation, thus providing extrahepatic tissues with the constituents for local GSH (re)synthesis. Hepatic GSH depletion occurs if the balance between synthesis, intracellular consumption and release of GSH cannot be maintained, and results in impaired antioxidant defense and attendant cell damage [7].

One of the processes in which GSH is involved, is the metabolism of the analgesic acetaminophen (APAP). At high doses, a significant fraction of APAP is metabolized by cytochrome P450s giving rise to the reactive compound N-acetyl-p-benzoquinone imine (NAPQI) and phenoxyl radicals derived thereof [8, 9]. NAPQI can be neutralized by GSH for subsequent export from the liver. Additionally, GSH reacts with the phenoxyl radical of APAP, resulting in the formation of a less reactive thiyl radical [10]. High doses of APAP may result in a drop in hepatic GSH levels and may cause acute liver failure [11].

Animal and in vitro studies showed that systemic ophthalmic acid (OPH) levels increased when hepatocellular glutathione and its constituent Z-cysteine, were depleted in APAP-induced hepatotoxicity models [12]. OPH is an endogenous tripeptide analogue of GSH and is formed by the same enzymes, with incorporation of 2-aminobutyric acid rather than Z-cysteine in the initial biosynthetic step [12-15]. OPH lacks a reactive thiol group and is thus devoid of antioxidant properties. It has been suggested that OPH makes use of the same transporter system as GSH and therefore would minimize cellular GSH efflux to preserve cell integrity [12]. Since L-cysteine availability is considered the rate- limiting factor in hepatic GSH formation, elevated plasma OPH concentrations may be a read-out of hepatic GSH depletion.

Patients undergoing partial hepatectomy for benign or malignant liver disease often receive APAP pre- and postoperatively to enhance their recovery through reduction of pain [16]. Reduced preoperative liver quality and chemotherapy, as well as extended resections can result in reduced liver function following resection. In the presence of a diminished liver volume and additional surgical stress during partial hepatectomy (PH), the administration of a normal dose of APAP has been suggested to lead to a faster depletion of hepatic GSH stores [17]. This is one of the reasons why APAP is considered contraindicated after hepatic resection by many clinicians. In the present study we investigated whether an APAP challenge resulted in altered plasma levels and liver content of GSH and OPH in patient groups undergoing abdominal surgery.

## 2. Materials and methods

### 2.1 Patient inclusion

All consecutive patients between October 2010 and October 2011 who were older than 18 years and underwent non-laparoscopic liver resection at the Maastricht University Medical Center for malignant disease, were considered for inclusion in this prospective study. In the same time frame, patients undergoing pancreatic surgery were included as control group with the following rationale: (I) they experience comparable surgical stress as patients undergoing liver resection but their functional liver capacity remains conserved, (II) there are no major differences in anesthetics during liver and pancreatic surgery, and (III) during pancreatic surgery blood can be drawn more easily from the portal and hepatic vein than during other types of major abdominal surgery allowing the study of splanchnic GSH/OPH release. Exclusion criteria in both groups were alcohol abuse up to six months before participation in this study, aberrations or insufficiency of kidney, liver, gut, heart or lungs, apart from the underlying malignancy, the presence of persistent inflammation in the gut or liver, the use of drugs known to affect liver metabolism, anemia or infection, HIV infection or hepatitis.

Patients were included at the outpatient department, and admitted to the hospital one day prior to operation. Routine blood tests were performed at this time. The study was approved by the medical ethical committee of the Maastricht University Medical Center (study number: NL26884.068.09 / 09-3-010) and conducted according to the revised version of the Declaration of Helsinki (October 2008, Seoul) and the Medical Research Involving Human Subjects Act (WMO). All patients participating in this study gave written informed consent. After surgery, standard clinical care was provided according to the enhanced recovery after surgery (ERAS) protocol [16].

### 2.2 Outcome and definitions

The primary aim of the study was to investigate whether systemic OPH levels could be an indicator of hepatic GSH depletion. Secondary outcome was the effect of an APAP challenge on generation of thiyl radicals. Liver resections were classified as major (>3 Couinaud segments) or minor (<3 segments) resections. Morbidity was defined as any complication within 90 days after surgery, whereas major morbidity comprised complications with a Dindo-Clavien grade of 3 or higher [18].

### 2.3 Operative procedure

Patients were anaesthetized using isoflurane and propofol. They routinely had an epidural catheter, urinary catheter, two peripheral venous catheters and catheters in a jugular vein and radial artery. Pylorus-preserving pancreaticoduodenectomy (PPPD; modified Whipple procedure) was performed for curation; in case of irresectability, a palliative double bypass was created. In this study, double bypass surgery and PPPD are both referred to as PPPD. PH was performed as detailed elsewhere [19].

### 2.4 Intravenous APAP-challenge model

**Figure 1.**
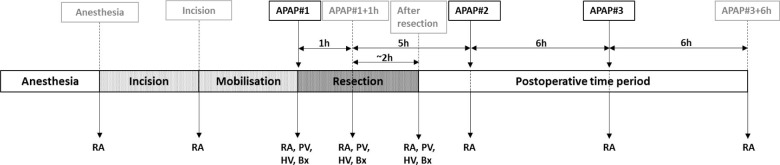
Timeline of sample collection and APAP-challenges. Study design with details on the timing of blood and tissue sampling, and the administration of three consecutive doses of APAP (APAP#1-3). The labels introduced in the top line are used throughout the manuscript. Abbreviations: RA, blood sample radial artery; PV, blood sample portal vein; HV, blood sample hepatic vein; Bx, liver biopsy; APAP, acetaminophen; h, hour.

APAP (1000 mg, Perfalgan solution for intravenous infusion, 10 mg/ml, Bristol-Myers Squibb Pharmaceuticals, no organic solvents added) was administered intravenously during surgery directly after mobilization of the liver but before resection (APAP#1), and six (APAP#2) and 12 (APAP#3) hours later (Figure 1). The APAP solution (100 mL) was administered in less than 5 minutes.

### 2.5 Blood and tissue sampling

Radial artery blood samples were obtained at eight predefined time points: after induction of anesthesia, after incision of the liver/pancreas, immediately before the first APAP challenge (APAP#1), one hour after the first APAP challenge (APAP#1+1h), and after resection of the liver/ pancreas (Figure 1). Postoperatively, radial artery blood samples were taken immediately before the second and third APAP challenge (APAP#2 resp. APAP#3), and six hours after the final challenge (APAP#3+6h). For study of venous- arterial differences (AVA), blood was drawn nearsimultaneously from the radial artery, portal vein, and one of the hepatic veins. This was performed on three occasions, *viz.* at APAP#1, APAP#1 + 1h, and after completion of liver/pancreatic resection. Concurrent intra-operative liver biopsies were taken in both groups on these time points. In case of liver resection, blood samples and biopsies were taken from the non-tumor bearing hemi-liver.

VA differences (AVA) across the portal drained viscera (PDV), liver and splanchnic area (SPL) were calculated using the following formulae:

AVAPDV = portal venous [X] - arterial [X]

Liver input = 0.30 * arterial [X] + 0.70 * portal venous [X]

ΔVALiver = hepatovenous [X] - liver input

ΔVASPL = hepatovenous [X] - arterial [X]

### 2.6 Sample preparation and OPH/GSH measurements

Blood samples were collected in pre-chilled heparinized vacuum tubes (6 mL) and immediately centrifuged at 4 °C at 3500 *g for 10 minutes in the operating theatre. Obtained plasma samples were stored at -80°C until analysis. For OPH and GSH measurements, plasma samples were deproteinized by the addition of an equal volume of a freshly prepared 5% (w/v) 5-sulphosalicylic acid solution containing 0.1% (w/v) vitamin C (British Drug Houses, Amsterdam, The Netherlands) to prevent oxidation of GSH. Liver samples were homogenized by microbeating 10 mg of liver tissue in 100 microliter of the above solution, and homogenates were stored at -80 °C until analysis. Prior to analysis, all samples were centrifuged for 10 minutes at 50000 *g at 4°C. OPH and GSH were measured in plasma and liver homogenate supernatants using liquid chromatography-mass spectrometry [20].

### 2.7 Quantification of thiyl radicals

Thiyl radicals were assayed using electron spin resonance (ESR) spectroscopy at low temperature [21]. For this, frozen human liver biopsies were placed in liquid nitrogen in a quartz liquid finger Dewar at the center of the spectrometer’s high sensitivity cavity. ESR spectra were recorded on an X-band spectrometer (Bruker EMX 1273, Biospin, Rheinstetten, Germany) operating at 9.50 GHz. Instrumental settings were: magnetic field: 3325 G; scan range: 150 G; modulation frequency 100 kHz modulation amplitude: 5 G; receiver gain: 1 x 10 [5]; power: 20 mW; time constant: 20.84 ms; scan time: 40.96 ms; number of scans: 20. Thiyl radicals were quantified by peak surface measurements using the WIN-EPR spectrum manipulation program (Version 2.11, Bruker, Rheinstetten, Germany).

### 2.8 Statistical analyses

Statistical analysis was performed using the Statistical Package for the Social Sciences (SPSS) version 24.0 (IBM, New York). All data are expressed as median with interquartile range. To compare categorical variables in the two surgical groups the Mann-Whitney *U* and Fisher’s Exact Test were applied where appropriate. Effects of the perioperative APAP challenge on circulating and hepatic GSH and OPH levels were analyzed using the Friedman test for repeated measurements. If appropriate, pre-defined pair-wise comparisons for circulating (baseline (=anesthesia) versus all other time points) and hepatic (baseline (=APAP#1) *versus* all other time points) analytes were made with a posthoc Wilcoxon signed-rank test and Bonferroni-Holm correction for multiple comparisons. Correlations between systemic GSH and OPH were evaluated with Spearman's rank test for nonparametric data. Venous-arterial gradients (ΔVA) of GSH and OPH were tested versus a theoretical median of zero using the Wilcoxon signed-rank test. A *p*-value <0.05 was considered statistically significant.

## 3 Results

### 3.1 Patient characteristics

Nineteen patients (seven females; 12 males) scheduled for a PPPD or liver resection were included in this study (Table 1). Seven patients had a pancreatic malignancy, of whom four underwent a PPPD and three received a palliative double bypass. None of these patients received neoadjuvant chemotherapy. Twelve patients underwent a liver resection for primary (n=2; isolated cases of intrahepatic cholangiocarcinoma and hepatocellular carcinoma) or secondary (n=10, nine cases of CRLM, single case of metastases of a melanoma) liver malignancies. Neoadjuvant chemotherapy was administered in six out of 12 patients undergoing liver resection. Three patients underwent major liver resection. Biochemical assessment showed no significant differences between the surgical groups in liver-related parameters (Table 1). Histopathological evaluation revealed that none of the patients had cirrhosis of the liver (a condition associated with reduced intrahepatic GSH levels) [22]. Regarding the postoperative course, no significant differences were observed between surgical groups with regard to length of hospital stay, overall and major morbidity (Table 1).

**Table:** 

	**Pancreatic resection group (PPPD) (n = 7)**	**Partial hepatectomy group (PH)(n = 12)**	**P-value**
*Gender*			
Male	3	9	1.000§
Female	4	3	
Median age (years)	67 [62-68]	65 [51-70]	0.592†
Median BMI	24.4 [23.1-27.1]	26.5 [24.4-30.8]	0.261†
*Preoperative laboratory values*			
AST (IU/L)	19 [17-24]	24 [19-27]	0.368†
ALT (IU/L)	34 [24-41]	29 [19-37]	0.659†
LDH (IU/L)	138 [122-154]	197 [157-268]	0.073†
GGT (IU/L)	75 [74-128]	66 [30-172]	0.659†
AP (IU/L)	138 [125-138]	112 [74-127]	0.100†
Bilirubin (micromol/L)	21 [18-45]	13 [10-19]	0.145†
Creatinine (micromol/L)	65 [61-76]	77 [67-92]	0.227†
*Preoperative chemotherapy*			
No	7	6	0.044§
Yes	0	6	
*Indication for surgery*			
Primary malignancy	7	2	n/a
Secondary malignancy	0	10	
*PVE*			
No	7	11	n/a
Yes	0	1	
*Pringle maneuver*			
No	7	8	n/a
Yes	0	4	
*Postoperative short-term outcome*			
Median hospital stay	12 [9-32]	9 [5-15]	0.227)
*Overall morbidity*			
No	1	7	0.147§
Yes	6	5	
*Major morbidity (DC III-V)*			
No	3	9	0.326
Yes	4	3	

Values depicted in median with interquartile range. §Fisher’s Exact test, )Mann-Whitney U test.

### 3.2 Effect of APAP challenge on arterial GSH and OPH levels

**Figure 2.**
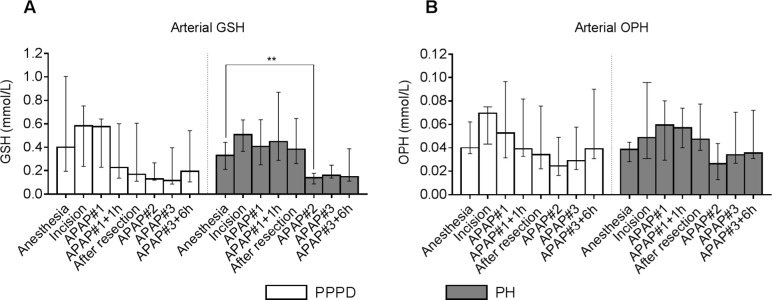
Ophthalmic acid and glutathione levels in arterial plasma during the course of the APAP challenges. Arterial GSH and OPH at consecutive time points in patients undergoing a PPPD (A) or PH (B). Data are plotted as median with interquartile range. *p**** < 0.05, **p**** < 0.01. Abbreviations: APAP, acetaminophen; PPPD, pylorus-preserving pancreatoduodenectomy; PH, partial hepatectomy; GSH, glutathione; OPH, ophthalmic acid.

Plasma levels of GSH and OPH were not different *(p* = 0.536 and *p* = 0.432, respectively) between surgical groups at baseline (i.e. time point of anesthesia) (Figure 2). A significant change in time was observed for plasma GSH levels in both surgical groups (*p* = 0.001 for both groups). Post-hoc pair wise comparisons of the respective time points versus baseline reached no significance in the PPPD group, whereas GSH levels were significantly time point, *p* = 0.003, Figure 2A). Plasma OPH levels changed significantly over time in patients undergoing PPPD (p = 0.013) or PH (p = 0.005), although the directionality in time was less clear than for GSH (Figure 2B). The latter appeared to be reflected in lack of significant changes in direct pair wise comparisons of time points versus baseline. Direct comparisons between the surgical groups revealed that neither GSH nor OPH levels differed significantly at any of the time points during the course of the APAP challenge (data not shown).

Similar directionality of correlations between arterial GSH and OPH was observed upon stratification for type of surgery (data not shown), hence data of all patients were pooled to increase power of correlation analysis. Arterial GSH and OPH were positively correlated at all time points (p between 0.510.90, supplemental data, Appendix 1).

### 3.3 Effect of APAP challenge on hepatic levels of GSH, OPH and APAP thiyl radicals

**Figure 3.**
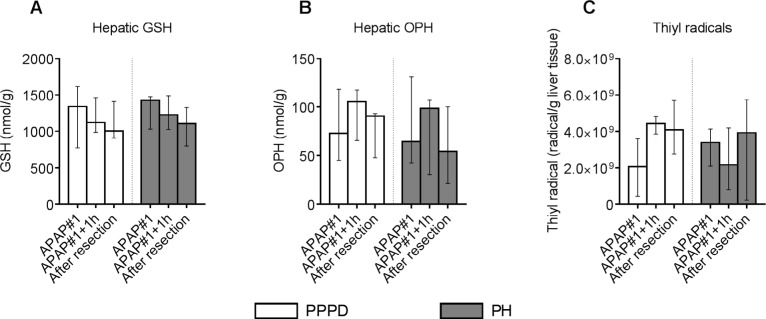
GSH, OPH and thiyl radicals in liver tissue during the course of the first APAP challenge GSH (A), OPH (B), and thiyl radical (C) levels measured at consecutive time points in homogenized liver biopsies of patients undergoing a PPPD or PH. Data are plotted as median with interquartile range. Abbreviations: APAP, acetaminophen; PPPD, pylorus-preserving pancreatoduodenectomy; PH, partial hepatectomy; GSH, glutathione; OPH, ophthalmic acid.

Hepatic GSH content prior to first APAP administration (APAP#1 time point) was similar in patients undergoing PPPD and PH ((1338 [769-1617] vs. 1425 [1030-1475] nanomol/g liver, resp.; *p* = 0.750) (Figure 3A). The median time between start (APAP#1) and end of resection was 122 [70-215] minutes, and did not differ between surgical groups *(p* = 0.145). Hepatic GSH did not change over time in patients undergoing PPPD *(p =* 0.779) *or* PH *(p =* 0.247).

Baseline hepatic OPH content was not different in patients undergoing PPPD or PH (73 [45-119] vs. 64 [42-131] nanomol/g liver, resp.; *p* = 0.892), and levels did not change during the course of APAP resection in either patients undergoing PPPD *(p* = 0.779) or PH *(p* = 0.247) (Figure 3B).

Likewise, baseline levels of APAP-derived thiyl radicals were similar in liver of patients undergoing PPPD or PH (2.110 [9] [0.36-5.610 [9]] vs. 3.410 [9] [0.11-6.710 [9]] radicals/g liver, resp.; *p* = 0.335), and levels did not change over time in either group *(p* = 0.717 and *p* = 0.867, resp.) (Figure 3C).

### 3.4 Effect of APAP no hepatic movement of GSH and OPH

Simultaneous drawing of portal venous, radial arterial and hepatic venous blood at three occasions during the course of the first APAP administration, allowed the assessment of the early effects of this challenge on the net extraction or net release of GSH and OPH by the liver (Appendix 2).

**Figure 4.**
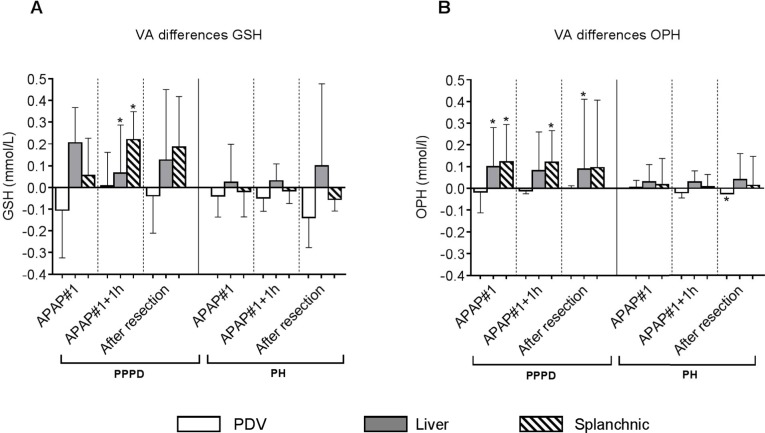
Venous-arterial differences across visceral tissues during the course of the first APAP challenge. Venous-arterial concentration gradients of GSH and OPH across the PDV, liver and splanchnic area in patients undergoing PPPD (A) or PH (B). Data are plotted as median with interquartile range. *p<0.05 versus a theoretical median of zero. Abbreviations: APAP, acetaminophen; PDV, portal drained viscera; PPPD, pylorus-preserving pancreatoduodenectomy; PH, partial hepatectomy; GSH, glutathione; OPH, ophthalmic acid.

Contrary to expectation, there was no net release of GSH from the liver at baseline in patients undergoing PPPD *(p* = 0.297) or PH *(p* = 0.677), although net release was apparent one hour after APAP administration in the PPPD group *(p* = 0.031) (Figure 4A, gray bars). In contrast, net hepatic and splanchnic release of OPH was observed at baseline and both time points after APAP challenge in patients undergoing PPPD *(p* between 0.016 and a trend of 0.063), but not in patients undergoing liver resection (Figure 4B).

VA differences for GSH across the PDV were not significant at baseline or the later time points in either group, indicating that there was no net movement of GSH across the tissues draining into the portal vein (Figure 4A, white bars). Likewise, in general there was no net movement of OPH across the PDV in the course of the first APAP administration, although net extraction by the PDV did occur after completion of liver resection *(p* = 0.031) (Figure 4B, white bars).

## 4 Discussion

In the present study we investigated whether plasma OPH is useful as a read-out for hepatic GSH depletion in humans. To this end, a total of three doses of APAP were administered in a peri-operative time frame of 18 hours, to patients with preserved (PPPD group) and reduced (PH group) liver mass. Our main finding is that the decline in plasma GSH, observed in both groups during the course of the APAP challenge, was not accompanied by a reciprocal increase in plasma OPH. Rather, the positive correlation between circulating GSH and OPH under a clinically realistic APAP regimen, calls for careful consideration of data from earlier animal and in vitro experiments.

Acute effects of the first gift of APAP on hepatic GSH homeostasis could be studied in the ˜3 hour interval between start and completion of the respective resection procedures. Within this time frame there were no alterations in hepatic GSH or OPH content, nor was there enhanced production of thiyl radicals in either patient group (Figure 3). Although the liver is considered the predominant source of GSH in the circulation [23], we did not observe net hepatic GSH release prior to, or after APAP administration in the present study (Figure 4). Net hepatic and splanchnic release of OPH was observed though in patients undergoing PPPD, with a similar magnitude maintained during the 3 hours after the first APAP administration. Above findings indicate that APAP did not result in acute oxidative stress or prompt alterations in hepatic GSH homeostasis. Obviously, longer term effects of the sequential APAP administrations could not be studied at the level of the liver. The integral APAP challenge resulted in significant lowering of circulating GSH levels (Figure 2). Plasma GSH levels were similar in both surgical groups at all studied time points. Hence, removal of part of the liver, considered the main source of circulating GSH, did not result in a further decline of plasma GSH. The latter is consistent with the absence of net hepatic GSH release in the current study.

Although animal studies revealed elevation of plasma OPH following APAP-induced depletion of hepatic and circulating GSH, an inverse relationship between plasma GSH and OPH was not apparent in our patients. The applied APAP doses in this study were equal to doses used in standard postoperative care and comparable with normal clinical practice (maximum of 4000 milligrams a day). Cumulative APAP dose was rather low in comparison to levels attained in in vitro models and mouse studies, the latter with concentrations up to 600 mg/ kg body weight [9, 12, 13]. Geenen et al. used a mathematical model based on data from hepatic cell lines to predict intracellular and extracellular concentrations of OPH following APAP administration [24]. Extracellular OPH concentrations remained stable until the intracellular GSH concentration decreased under a threshold, after which OPH production increased. Based on above studies it can be concluded that OPH is a good marker for hepatic GSH homeostasis under conditions of severe GSH depletion. Unchanged intra-operative hepatic content of GSH, OPH, and APAP-derived thiyl radicals indicate that those conditions were not met in the current human model, at least not in the first three hours after the initial APAP dose. Assuming an average adult liver weight of 1500 g, and conversion of 5% of ingested APAP (i.e. 50 mg = 0.33 mmoL) into GSH-consuming NAPQI, we estimated that the initial APAP dose resulted in consumption of 0.22 pmol GSH/g liver. Considering hepatic GSH levels of 1.5 pmol GSH/g liver (Figure 3A), about 15% of hepatic GSH content would be consumed. This is far less than the hepatic GSH depletion (>90%) after an APAP overdose in mice [12]. Increasing the APAP dose would not be justified because of concerns of acute liver failure [25,26], especially for patients undergoing (liver) surgery.

Arterial GSH and OPH were positively correlated at all studied time points. Since OPH and GSH are synthesized through the same enzymatic machinery [12], is it likely that there is competition between the initial substrates which may explain the same dynamics in plasma. In the present study,hepatic GSH and OPH levels did not correlate with their respective levels in plasma (p between 0.071-1.000). This is in contrast with findings of Soga et al. who showed a good correlation between hepatic and systemic OPH in an APAP-related mouse model [12] and demonstration by Kombu et al. that ^2^H labeling of plasma GSH was an indicator for ^2^H labeling of liver GSH in a rat model [14]. An explanation could be that in the rat model of Kombu et al. arterial blood sampleswere taken from the aorta, whereas in the mouse model of Soga et al. it is unclear from what puncture site blood samples were taken. Geenen et al. measured OPH concentrations in medium of cultured hepatic cell lines [27]. All studies thus have potentially assessed OPH concentrations in different fluid compartments, and the influence of this on the results is unclear.

The fact that APAP administration during liver surgery did not lead to (immediate) GSH depletion or increased OPH levels granted valuable information about the safety of administration of APAP used after liver surgery in a standard postoperative care program. Based on stable levels of hepatic GSH, OPH and thiyl radicals during surgery, standard APAP administration seems to be safe in this specific population with regards to GSH homeostasis. Hence, our findings support the notion of Hughes et al. who concluded that use of APAP is safe in patients undergoing major liver resection, provided that liver function is adequate [28]. However, no general statements can be made on the basis of the current experiment, since GSH homeostasis and susceptibility to xenobiotic toxicity are influenced by numerous factors including genetic polymorphism in glutamate cysteine ligase [29], altered levels of the expression of genes encoding the y-glutamyltranspeptidase enzyme, and GSH synthase deficiency and changes in methionine metabolic pathway (i.e. in cirrhotic patients and in patients with homocysteinemia) [30]. Even in healthy individuals, peak ALT elevations up to 8-fold were reported in 27% of participants receiving a therapeutic dose of APAP [31]. Therefore, caution is still warranted with APAP as a postoperative analgesic following liver resection.

The present study is hampered by some limitations. The APAP solution that was used in this study contained 0.1 mg/ mL *L-cysteine.* Although this could have effected GSH/OPH synthesis, this amount is 80 times lower on a molar basis than the amount of APAP administered. In patients suffering from APAP-intoxication, the amount of *L*-cysteine that is repeatedly administered is more than 1000 times higher. In addition, only three patients underwent major hepatectomy. It was therefore impossible to determine the effect of liver resection volume on arterial or hepatic GSH and OPH. At last, the influence of anesthesia on GSH and OPH metabolism is unknown, and it would be worthwhile to assess arterial OPH and GSH before induction of anesthesia as optimal baseline measure.

In conclusion, this is the first human study in which the usefulness of OPH as a read-out for hepatic GSH metabolism was explored. APAP administration had no acute effects on hepatic levels of GSH and OPH, and eventually resulted in lowering of GSH in the circulation. Plasma GSH and plasma OPH were positively correlated at all time points during the course of the APAP challenge, and raises the question whether hepatic GSH homeostasis was sufficiently challenged in the current study. Alternatively, findings from animal studies may be explained by APAP dosing effects. Future studies are needed in order to examine validity of plasma OPH as a biomarker for hepatic GSH depletion in clinical practice. Informative patient groups may be patients with acute (APAP intoxication) or postresectional liver failure [32].

## References

[1] van den Broek MA, Olde Damink SW, Dejong CH, Lang H, Malago M, Jalan R, Saner FH (2008). Liver failure after partial hepatic resection: definition, pathophysiology, risk factors and treatment.. Liver Int.

[2] Hoekstra LT, de Graaf W, Nibourg GA, Heger M, Bennink RJ, Stieger B, van Gulik TM (2013). Physiological and biochemical basis of clinical liver function tests: a review.. Ann Surg.

[3] van Mierlo KM, Schaap FG, Dejong CH, Olde Damink SW (2016). Liver resection for cancer: New developments in prediction, prevention and management of postresectional liver failure.. J Hepatol.

[4] Hinson JA, Reid AB, McCullough SS, James LP (2004). Acetaminophen-induced hepatotoxicity: role of metabolic activation, reactive oxygen/nitrogen species, and mitochondrial permeability transition.. Drug metabolism reviews.

[5] Biolo G, Antonione R, De Cicco M (2007). Glutathione metabolism in sepsis.. Crit Care Med.

[6] Hammond CL, Lee TK, Ballatori N (2001). Novel roles for glutathione in gene expression, cell death, and membrane transport of organic solutes.. J Hepatol.

[7] Schauer RJ, Gerbes AL, Vonier D, Meissner H, Michl P, Leiderer R, Schildberg FW, Messmer K, Bilzer M (2004). Glutathione protects the rat liver against reperfusion injury after prolonged warm ischemia.. Ann Surg.

[8] Jaeschke H, McGill MR, Ramachandran A (2012). Oxidant stress, mitochondria, and cell death mechanisms in drug-induced liver injury: lessons learned from acetaminophen hepatotoxicity.. Drug metabolism reviews.

[9] McGill MR, Lebofsky M, Norris HR, Slawson MH, Bajt ML, Xie Y, Williams CD, Wilkins DG, Rollins DE, Jaeschke H (2013). Plasma and liver acetaminophen-protein adduct levels in mice after acetaminophen treatment: dose-response, mechanisms, and clinical implications.. Toxicol Appl Pharmacol.

[10] Ramakrishna Rao DN, Fischer V, Mason RP (1990). Glutathione and ascorbate reduction of the acetaminophen radical formed by peroxidase. Detection of the glutathione disulfide radical anion and the ascorbyl radical.. J Biol Chem.

[11] Jaeschke H, Williams CD, Ramachandran A, Bajt ML (2012). Acetaminophen hepatotoxicity and repair: the role of sterile inflammation and innate immunity.. Liver Int.

[12] Soga T, Baran R, Suematsu M (2006). Differential metabolomics reveals ophthalmic acid as an oxidative stress biomarker indicating hepatic glutathione consumption.. J Biol Chem.

[13] Abbas R, Kombu RS, Ibarra RA, Goyal KK, Brunengraber H, Sanabria JR (2011). The Dynamics of Glutathione Species and Ophthalmate Concentrations in Plasma from the VX2 Rabbit Model of Secondary Liver Tumors.. HPB Surg.

[14] Kombu RS, Zhang GF, Abbas R (2009). Dynamics of glutathione and ophthalmate traced with 2H-enriched body water in rats and humans.. Am J Physiol Endocrinol Metab.

[15] Dello SA, Neis EP, de Jong MC (2012). Systematic review of ophthalmate as a novel biomarker of hepatic glutathione depletion. Clin Nutr.

[16] van Dam RM, Hendry PO, Coolsen MM (2008). Initial experience with a multimodal enhanced recovery programme in patients undergoing liver resection.. Br J Surg.

[17] Abu-Amara M, Yang SY, Tapuria N, Fuller B, Davidson B, Seifalian A (2010). Liver ischemia/reperfusion injury: processes in inflammatory networks--a review.. Liver Transpl.

[18] Dindo D, Demartines N, Clavien PA (2004). Classification of surgical complications: a new proposal with evaluation in a cohort of 6336 patients and results of a survey.. Ann Surg.

[19] Dejong C, Garden O, AA Kingsnorth A (2003). Neoplasms of the liver. In: Majid. Advanced surgical practice. London: Greenwich medical Media.

[20] Dello SA, van Eijk HM, Neis EP, de Jong MC, Olde Damink SW, Dejong CH (2012). Ophthalmate detection in human plasma with LC-MS-MS.. J Chromatogr B Analyt Technol Biomed Life Sci.

[21] Sevilla MD, Becker D, Swarts S, Herrington J (1987). Sulfinyl radical formation from the reaction of cysteine and gluthathione thyil radicals with molecular oxygen.. Biochemical and Biophysical research Communications.

[22] Czeczot H, Scibior D, Skrzycki M, Podsiad M (2006). Glutathione and GSH-dependent enzymes in patients with liver cirrhosis and hepatocellular carcinoma.. Acta Biochim Pol.

[23] Lauterburg BH, Adams JD, Mitchell JR (1984). Hepatic glutathione homeostasis in the rat: efflux accounts for glutathione turnover.. Hepatology.

[24] Geenen S, du Preez FB, Snoep JL (2013). Glutathione metabolism modeling: a mechanism for liver drug-robustness and a new biomarker strategy.. Biochim Biophys Acta.

[25] Ostapowicz G, Fontana RJ, Schiodt FV (2002). Results of a prospective study of acute liver failure at 17 tertiary care centers in the United States.. Ann Intern Med.

[26] Craig DG, Lee A, Hayes PC, Simpson KJ (2010). Review article: the current management of acute liver failure.. Aliment Pharmacol Ther.

[27] Geenen S, Michopoulos F, Kenna JG, Kolaja KL, Westerhoff HV, Wilson I (2011). HPLC-MS/MS methods for the quantitative analysis of ophthalmic acid in rodent plasma and hepatic cell line culture medium.. J Pharm Biomed Anal.

[28] Hughes MJ, Harrison EM, Jin Y, Homer N, Wigmore SJ (2015). Acetaminophen metabolism after liver resection: A prospective case- control study.. Dig Liver Dis..

[29] Walsh AC, Feulner JA, Reilly A (2001). Evidence for functionally significant polymorphism of human glutamate cysteine ligase catalytic subunit: association with glutathione levels and drug resistance in the National Cancer Institute tumor cell line panel.. Toxicol Sci.

[30] Lu SC (1999). Regulation of hepatic glutathione synthesis: current concepts and controversies.. FASEB J.

[31] Watkins PB, Kaplowitz N, Slattery JT (2006). Aminotransferase elevations in healthy adults receiving 4 grams of acetaminophen daily: a randomized controlled trial.. JAMA.

[32] Donnelly MC, Davidson JS, Martin K, Baird A, Hayes PC, Simpson KJ (2017). Acute liver failure in Scotland: changes in aetiology and outcomes over time (the Scottish Look-Back Study).. Aliment Pharmacol Ther.

